# Novel Drugs for the Management of Diabetes Kidney Transplant Patients: A Literature Review

**DOI:** 10.3390/life13061265

**Published:** 2023-05-26

**Authors:** Nancy Daniela Valencia-Morales, Beatriz Rodríguez-Cubillo, Rómulo Katsu Loayza-López, Maria Ángeles Moreno de la Higuera, Ana Isabel Sánchez-Fructuoso

**Affiliations:** Nephrology Department, Hospital Clínico de Madrid, 28040 Madrid, Spain

**Keywords:** diabetes, kidney transplant, glucagon-like peptide 1 receptor agonists, sodium-glucose cotransporter type 2 inhibitors

## Abstract

The management of diabetes and renal failure is changing thanks to the appearance of new drugs such as glucagon-like peptide 1 receptor agonists (GLP1-RA) and sodium-glucose cotransporter type 2 inhibitors (SGLT2i) that have benefits in terms of survival and cardiorenal protection. Based on the potential mechanisms of GLP1-RA, kidney transplant recipients (KTRs) could benefit from their effects. However, high-quality studies are needed to demonstrate these benefits, in the transplant population, especially those related to cardiovascular benefits and renal protection. Studies with SGLT2i performed in KTRs are much less potent than in the general population and therefore no benefits in terms of patient or graft survival have been clearly demonstrated in this population to date. Additionally, the most frequently observed side effects could be potentially harmful to this population profile, including severe or recurrent urinary tract infections and impaired kidney function. However, benefits demonstrated in KTRs are in line with a known potential effects in cardiovascular and renal protection, which may be essential for the outcome of transplant recipients. Better studies are still needed to confirm the benefits of these new oral antidiabetics in the renal transplant population. Understanding the characteristics of these drugs may be critical for KTRs to be able to benefit from their effects without being damaged. This review discusses the results of the most important published studies on KTRs with GLP1-RA and SGLT2i as well as the potential beneficial effects of these drugs. Based on these results, approximate suggestions for the management of diabetes in KTRs were developed.

## 1. Introduction

Chronic kidney disease (CKD) is a common and serious health problem that affects millions of people worldwide. According to epidemiological data, CKD is becoming more prevalent, with an estimated 10% of the global population affected [1]. The incidence of CKD is increasing due to several factors, including an aging population, the growing burden of chronic diseases such as diabetes mellitus and hypertension, and lifestyle factors such as poor diet and lack of exercise. CKD is associated with significant morbidity and mortality, and it is a major risk factor for cardiovascular disease [2]. The main cause of death in patients with end-stage kidney disease is cardiovascular events, regardless of whether patients are in dialysis, including after renal transplantation [3].

**Figure 1:**
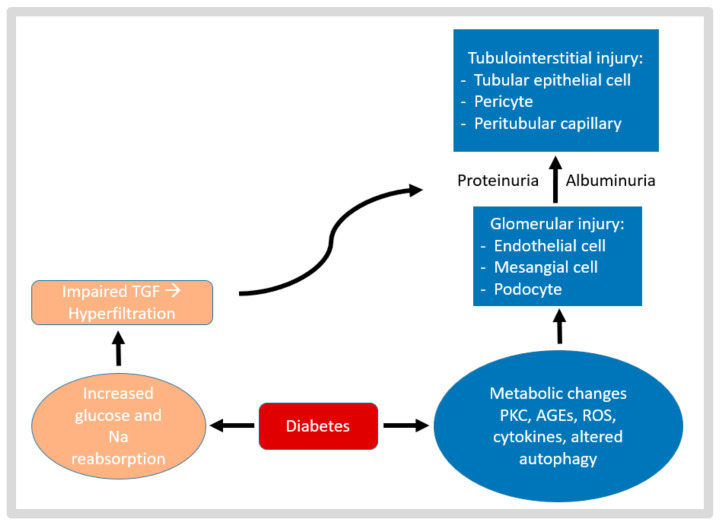
The pathophysiological mechanism of diabetic kidney injury. Diabetes-induced hyperglycemia, hypertension, high uric acid levels, and dyslipidemia cause PKC activation, oxidative stress, increased cytokine levels, and altered autophagy in glomerular cells and proximal tubular epithelial cells, as well as hemodynamic disruption (altered tubuloglomerular feedback, TGF), resulting in glomerular and tubulointerstitial injury. AGEs, advanced glycation end products; PKC, protein kinase C; ROS, reactive oxygen species; TGF, tubuloglomerular feedback.

Kidney transplantation is a life-saving treatment for patients with end-stage kidney disease. According to the latest data report of the US transplant registry, almost 47% of patients on the kidney transplant waiting list had diabetes mellitus and trend forecast that this proportion will increase [4]. Furthermore, 15–30% of kidney transplant recipients (KTRs) without diabetes developed persistent hyperglycemia for more than 6 weeks following a transplant, which is known as post-transplant diabetes mellitus (PTDM) [5]. This situation gives rise to a high prevalence of individuals with both a kidney transplant and pre-existing diabetes or PTDM. Some mechanisms explain how diabetes mellitus affects the kidneys both pre- and post-transplantation, as shown in Figure 1.

PTDM is a common and serious complication that occurs in a significant proportion of KTRs. According to a 5-year follow-up multicenter cohort study of KTRs who received deceased donor kidneys, PTDM was often observed with a cumulative prevalence of close to 30% [6]. The first 3 months following transplantation constitute the period with the highest incidence of PTDM [7].

In 2014, an international consensus meeting defined the term PTDM and clarified the definition of the term “new-onset diabetes after transplantation (NODAT)” with the outcome of rectifying the mistake to include patients who may have had undiagnosed diabetes before transplantation [8,9]. As some transplant centers do not test for undiagnosed diabetes during the pretransplant examination, pretransplant diabetes is not accurately diagnosed, making NODAT entity certification challenging.

Risk factors for PTDM include older age, a family history of diabetes, obesity, and pre-existing diabetes or impaired glucose tolerance [10,11,12,13], some of which may be related to the kidney transplant (Table 1).

The principal risk factors specifically associated with transplantation are viral infections and immunosuppressive therapy. It is generally recognized that corticosteroids can lead to hyperglycemia and increase the risk of developing diabetes. Impaired insulin sensitivity, increased hepatic gluconeogenesis, and hunger stimulation with consequent weight gain are some of the factors driving corticosteroid-induced diabetes [10]. Tacrolimus and cyclosporin are well known to increase the risk of blood glucose parameters, risk of PTDM, and cardiovascular disease [12]. Studies using pancreatic histology sections from both animals and people have shown that taking calcineurin inhibitors (CINs) causes islet cell apoptosis to rise and beta-cell mass to decrease [14]. PTDM incidence and cardiovascular risk are more strongly correlated with tacrolimus; even the serum concentration levels >10 ng/mL during the first 3 months post-transplantation predispose to high glucose levels [10]. Another option in immunosuppressive therapy related to PTDM is the mammalian target of rapamycin (mTOR) inhibitors. Most studies were developed with sirolimus, and have described that the risk may be increased by hypertriglyceridemia and hyperinsulinemia by producing insulin resistance and affecting the beta-cell mass and function [14,15].

PTDM is associated with increased morbidity and mortality, and it can also contribute to the development of cardiovascular disease and other complications [9,16,17]. The management of diabetes mellitus is an important area of care for KTRs. Additionally, the early detection and management of PTDM can help reduce the risk of complications and improve patient outcomes. One of the most crucial factors is the rapid identification of diabetic nephropathy in PTDM patients following kidney transplant, which is connected to kidney allograft failure [18].

In this narrative review, we summarize the published literature about the management of diabetes in renal transplants and the use of new agents, namely glucagon-like peptide 1 receptor agonists (GLP1-RA) and sodium-glucose cotransporter type 2 inhibitors (SGLT2i), in the kidney transplant field. The most relevant case series, retrospective and prospective studies, as well as clinical trials published to date were included, with the main objective of evaluating the impact of these drugs in the renal transplant population.

## 2. Recommendations for the Management of Diabetes in Renal Transplant Patients

Recommendations for the management of hyperglycemia and diabetes in KTRs are based on guidelines and expert consensus [5,6,7,19,20,21,22,23,24], which do not clarify the most appropriate treatment for these patients. These recommendations include a multifactorial approach which includes lifestyle changes, pharmacological therapy, and the close surveillance of blood glucose levels [7,9,25]. Lifestyle interventions have been studied in KTRs, but without clear results in terms of improving metabolic parameters or long-term positive results [25].

Insulin therapy is currently considered the first-line treatment in hyperglycemia immediately following transplantation [26], given the rapid changes in glucocorticoid doses and allograft function. Moreover, the strict monitoring of blood glucose after transplantation has been shown to reduce the risk of developing a sustained PTDM [26]. However, in more stable patients, oral drugs are preferred, which, in addition to avoiding needlesticks, may have other cardiovascular and renal protective effects [22,27].

Metformin remains a fundamental pillar for the management of diabetes in the general population, and its use is increasingly encouraged in the kidney transplant population [28]. Several studies have shown the safety of its use in stable patients with preserved renal function. Recent guidelines [5,29] have promoted metformin as first-line oral treatment in KTRs with an estimated glomerular filtration ratio (eGFR) of ≥30 mL/min/1.73 m^2^ and BMI ≥ 25 kg/m^2^.

Gliptins inhibit the degradation of incretin hormones, including glucagon-like peptide 1 (GLP-1) and glucose-dependent insulinotropic polypeptide, by inhibiting dipeptidyl peptidase-4 (DDP-4) enzymes. This results in increased insulin synthesis and secretion, the suppression of glucagon secretion, the inhibition of gastric emptying, and the suppression of appetite and dietary intake [21]. Some favorable characteristics of gliptins including its weight neutrality, low risk of hypoglycemia, and low risk of drug–drug interactions. As linagliptin does not interact with the CIN or mTOR inhibitors, nor precise adjustment renal function, it is quite safe to use in the renal transplant recipient [30,31]. Additionally, DDP-4 inhibitors have theoretically been shown to protect the pancreatic beta-cells by activating GLP-1, which is why it has been proposed as an interesting drug in the recent transplant period [32].

**Figure 2:**
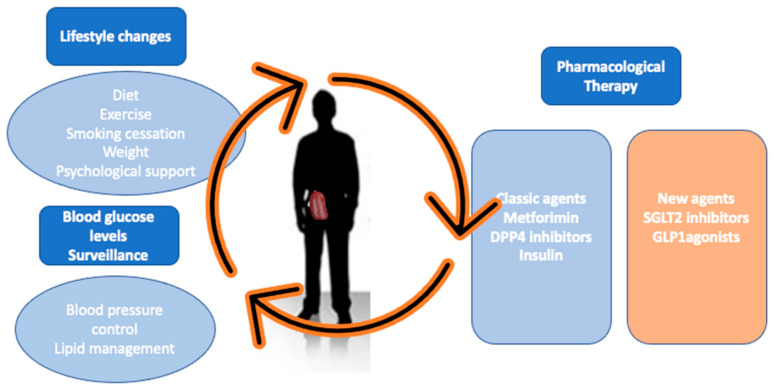
Challenges in diabetes with KTR. The most important challenges in the management of a transplant patient with diabetes include: to achieve improved graft and patient survival as well as minimize cardiovascular events, which are frequently associated with PTDM. To achieve this, our goal is to ensure that the patient has a healthy lifestyle, which includes avoiding toxic habits, diet, and exercise, as well as the good control of blood glucose, blood pressure, and lipid profile. We have a range of therapies that, in addition to helping to control blood glucose, could also be effective in controlling the diet, weight, and blood pressure, and even provide renal and cardiovascular protection benefits.

However, it has been shown that diabetic nephropathy can occur after kidney transplantation, despite intensive glycemic control [33,34]. Moreover, these drugs do not have the added benefit of cardiorenal protection present in other new antidiabetic drugs, such as GLP1-RA or SGLT2i [31]. Thus, it will probably be considered a second-line drug in the coming years, limited to patients with intolerance to other more potent drugs or with broader benefits (Figure 2).

## 3. New Antidiabetics in Renal Transplant GLP1-Ras

### 3.1. Evidence and Demonstrated Benefits of GLP1-RA Use in General and Renal Transplant Populations

GLP-1 is a natural incretin hormone that is swiftly produced after the intake into the bloodstream by enteroendocrine L-cells in the colon and distal small intestine. The many effects of GLP1-RA include increased insulin secretion and sensitivity, decreased hepatic gluconeogenesis, delayed stomach emptying, and reduced central appetite centers [35]. Several meta-analyses have demonstrated that GLP1-RA agents reduce the risk of cardiovascular events in patients with type 2 diabetes mellitus [36,37]. Moreover, beyond the benefits associated with glycemic control, these drugs have shown a protective effect at the cardiovascular level in diabetic patients [31].

In relation to kidney outcomes, the AWARD-7 trial was the first to demonstrate that dulaglutide delays the decline in rate eGFR compared to insulin glargine in patients with type 2 diabetes mellitus and moderate to severe CKD at 52 weeks follow-up [38]. Both the LEADER and SUSTAIN trials showed improvements in albuminuria, employing liraglutide and semaglutide, respectively [39]. The LEADER trial also showed that liraglutide, as dulaglutide, decreased the development and progression of diabetic kidney disease when compared with the placebo.

The evidence of the use of GLP1-RA in KTRs to date is limited as the published studies include few patients and show limited statistical power in demonstrating the benefits. Table 2 includes all actual studies of GLP1-RA in solid organ transplant (SOT) recipients with data about glycemic control, body weight, kidney function, interaction with immunosuppressive treatment, and side effects. The studies included in this review have a mixture of different SOTs given the lack of studies with a significant number of KTRs.

Sweiss et al. led the largest study in terms of the number of cases, including 83 KTRs treated with GLP1-RA (liraglutide, dulaglutide, semaglutide, exenatide) [50]. A statistically significant difference in median fasting blood glucose, glycated hemoglobin (HbA1c), and weight loss at baseline to 3–12 months (*p* < 0.0001) was observed. Singh et al., in a retrospective study that included 81 KTRs, compared the action of liraglutide and dulaglutide [44]. The results were more favorable for the use of dulaglutide, in terms of weight reduction (−5.2%, *p* < 0.03) and HbA1C (−8.4%, *p* < 0.49).

Regarding kidney function, most studies have failed to demonstrate benefits after the start of GLP1-RA [23,49,51] except for three studies: Singh et al. showed the benefits of the use of dulaglutide (improved eGFR of 15%) [44]; Sweiss et al. observed an improvement of 5 mL/min eGFR (*p* < 0.0001) [50] and Vigara et al. also found a modest benefit of GPL1-RA use on renal function (+3.5 mL/min of eGFR, *p* < 0.3) [49].

The potential interactions between GLP1-RA and tacrolimus were investigated. Cytochrome P450 enzymes and transporter-mediated drug–drug interactions are not involved in the metabolism of GLP1-RA, as this was eliminated by proteolytic degradation [51]. Hence, there is a low likelihood of an interaction between concurrent tacrolimus treatments. However, another possible factor to take into account for GLP1-RA in immunosuppressive treatment could be the interaction in their absorption, due to the effect of a delay in stomach emptying [52]. The investigation performed by Pinelli et al. [40] measured the tacrolimus area under the curve before and after the administration of a 21-day course of liraglutide, and no significant changes in tacrolimus blood concentrations were observed. Moreover, Thangavelu et al., in a retrospective study, analyzed the impact of exenatide, liraglutide, dulaglutide, or semaglutide on tacrolimus levels, without finding significant alterations [45]. However, further studies are still needed to conclude this field.

The most frequent side effects of GLP1-RA described in SOT recipients are gastrointestinal, including nausea, diarrhea, vomiting, and abdominal pain. However, Kim et al. found an improvement in gastrointestinal problems in 42.9% of patients who developed any symptom after 3 months [48]. Despite all the above, there is no evidence of long-term adverse effects as the longest follow-up in the series lasted 28 months [46]. It is therefore essential to appropriately select patients to avoid oral intolerance [31], especially in a patient population already affected by gastrointestinal adverse effects associated with other drugs, primarily mycophenolate.

Finally, GLP1-RA could associate hypoglycemia, as demonstrated in the general population, in approximately 12.3% with dulaglutide [52]. In studies of KTRs, hypoglycemia was only found in the series of Singh et al. and Kim et al. (Table 2). However, hypoglycemic events are presumably related to the concomitant use of other hypoglycemic agents, such as insulin. In this context, the use of GLP1-RA has been related to the reduction of 16.25 units of insulin per year [48] and the reduction in the doses of other oral antidiabetic drugs [44,45,48].

### 3.2. Potential Benefits of the Use of GLP1-RA in General and Renal Transplant Populations

**Figure 3:**
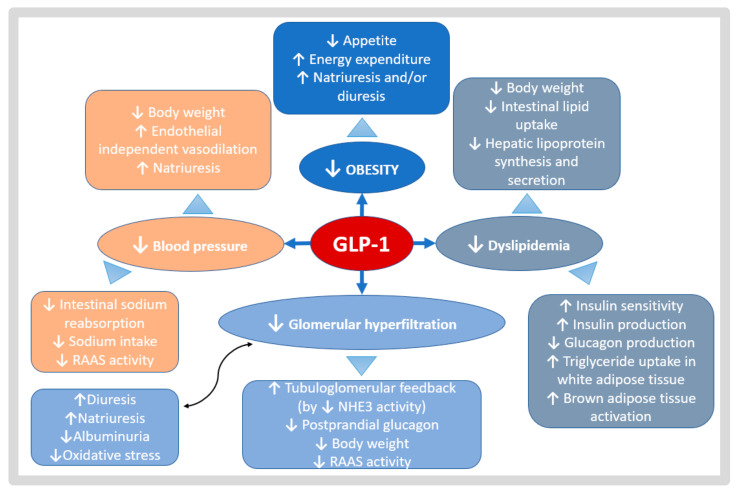
Potential benefits of GLP1-RA in CKD and KTR. GLP1-RA effects act in different metabolic pathways. A gut–renal axis is possible, with regulatory linkages through the gastrointestinal tract, central nervous system, and kidney. The reduction in weight (effects represented in deep blue), reduction in lipid levels (effects represented in gray), reduction in glomerular hyperfiltration (effects represented in sky-blue), and better blood pressure control (effects represented in orange), may be the principal outcomes of the physiological mechanisms of GLP1-RA.

There are several mechanisms attributed to GLP1-RA that could be renal and kidney graft-protective, such as the reduction in intraglomerular pressure, the induction of natriuresis, as well as the regulation of the immune system, inflammation, and Redox system [53]. Moreover, GLP1-RA has been shown to have anti-atherogenic properties [54] and could contribute to the mitigation of tacrolimus-induced toxicity in pancreatic beta-cells [55]. These effects could explain the cardiovascular protective benefits attributed to these drugs beyond glycemic or metabolic control. KTRs have a complication profile that could certainly benefit from these potential effects, which are summarized in Figure 3. All of the great benefits may be in part ascribed to a beneficial balance between oxidant and antioxidant pathways that are associated with anti-inflammatory or antifibrotic effects, or even an erythropoiesis stimulation.

The mechanisms of action of GLP-1RA in the kidney are not completely understood but may involve both neural and nonneural pathways. A gut–renal axis is possible, with regulatory linkages through the gastrointestinal tract, central nervous system, and kidney. The main physiological effect of GLP1-RA on the kidney may be to reduce prandial intraglomerular pressure to reduce the macronutrient (glucose, amino acids, and free fatty acids) loss in the glomerular filtrate. This would allow increased time for macronutrient uptake by other tissues without having to expend energy to transport macronutrients back into the body through the proximal tubule or overwhelming the proximal tubule reuptake system for macronutrients. It may do so by decreasing the sympathetic activity at the glomerulus through the central nervous system or by direct effects on the mesangium and renal interstitium [56,57].

Another one of the most important effects is that of GLP1-RA-induced natriuresis. GLP-1 and GLP1-RA have also been shown to reduce NHE3 (sodium-hydrogen antiporter 3) dependent proximal tubule sodium reabsorption in both animals and humans [58]. The inhibition of NHE3 activity in the proximal tubule would increase sodium transport from the distal tubule to the macula densa in the kidney. This would lead to the inhibition of the tubular glomerular feedback, which would decrease the intraglomerular pressure, hyperfiltration, and renin–angiotensin system activity [23]. It would be assumed that lowering intraglomerular pressure would have an antiproteinuric impact on diabetic kidneys and aid in maintaining kidney function [23].

Additionally, GLP1-RA could exhibit a specific contribution to regulating the immune system and inflammation [50]. Several molecular pro-inflammatory players such as oxidative stress, immune cell recruitment, cytokines production, and lipotoxicity, might be modulated by GLP1-RA [59]. In vitro studies have shown that GLP1-RA could suppress the macrophage secretion of different inflammatory cytokines (IFN-γ, IL-17, IL-2, TNF-β, IL-6, IL-1β) but could increase the anti-inflammatory cytokine (IL-10) [50]. Furthermore, in studies performed in murine models, it has been observed that GLP1-RA might be involved in the reduction in inflammatory cytokines, monocyte migration, and infiltration as well as in the increment in T-regulated cells [53].

Moreover, GLP1-RA has been shown to have anti-atherogenic properties, also presumably via indirect effects on lipids, glucose, weight, and blood pressure as well as indirect effects on inflammation and ischemia [54]. The prevention of glomerular atherosclerosis, which shares macrovascular disease’s predictors and mediators, may be aided by similar actions.

Regarding the specific benefits of these drugs in KTRs, a protective effect of GLP1-RA against toxicity caused by the most common immunosuppressant has been described. Tacrolimus and sirolimus treatment are toxic to pancreatic cells. A study performed by Dai et al. with human islets transplanted into immunodeficient mice treated with tacrolimus or sirolimus demonstrated that the treatment with both drugs reduced beta-cell granules, increased islet amyloid deposition, increase beta-cell apoptosis, and autophagy in the pancreatic islet cell [55]. Additionally, in pancreatic beta-cells, calcineurin/NFAT signaling is thought to positively regulate insulin secretion and, in juvenile human islets, beta-cell proliferation [60]. Thus, the inhibition of this pathway (via tacrolimus) could be toxic to the production of both, insulin, and beta-cells [61]. Additionally, mTOR signaling is crucial in many cells, including pancreatic beta-cells, as well as in the cellular response to signals. Sirolimus treatment disrupts the insulin signal transduction, causing insulin resistance, reducing insulin secretion, and decreasing beta-cell survival and proliferation [55].

Interestingly, GLP1-RA could have therapeutic effects on tacrolimus and sirolimus-induced pancreatic beta-cell injury. In this regard, Lim et al. observed that rats with tacrolimus-induced diabetes mellitus exhibited increased autophagy-associated protein expression and autophagic vacuole numbers in pancreatic beta cells [62]. GLP1-RA cotreatment decreased the tacrolimus-induced hyperglycemia, oxidative stress, and apoptosis, accompanied by decreased autophagy-associated protein expression and autophagosome numbers. In addition, Dai et al. demonstrated that the treatment with a GLP1-RA completely prevented the tacrolimus-induced beta-cell dysfunction and partially prevented sirolimus-induced beta-cell dysfunction [55]. These results highlight the importance of both calcineurin and mTOR signaling in normal human beta-cell function in vivo and suggested that the modulation of these pathways may prevent or ameliorate PTDM.

Based on the potential mechanisms of these drugs, kidney transplant patients could benefit from their effects. However, high-quality studies are needed to demonstrate these benefits, especially analyzing cardiovascular outcomes in the transplant population, and particularly those related to cardiovascular benefits and renal protection.

## 4. New Antidiabetics in Renal Transplant SGLT2 Inhibitors

### 4.1. Evidence and Demonstrated Benefits of SGLT2i Use in General and Renal Transplant Populations 

SGLT2i were originally developed to treat type 2 diabetes, and have demonstrated protective cardiorenal benefits in the general population with diabetes and patients with known kidney disease [63,64,65,66,67]. The main mechanism of action of SGLT2i is to inhibit glucose and sodium reabsorption at the brush border of the renal proximal tubules. However, benefits are increasingly being attributed to these drugs that concern not only the control of metabolic syndrome, but also the reduction in oxidative stress, inflammation, and fibrosis [68,69].

The beneficial effects of these drugs related to renal function consist of decreasing proteinuria, slowing the rate of development of end-stage renal disease, and the need to initiate renal replacement therapy. In addition, it has also been shown that the use of these drugs can reduce cardiovascular events and cardiovascular mortality in patients with CKD [64,70,71], regardless of underlying diabetes [72]. Based on these study results, SGLT2i have emerged even as a new treatment option for CKD [73,74], considering that they are indicated for the slow progression of the disease [22,75]. Renal transplant patients could also benefit from these effects [75], and studies are underway to analyze whether these similar cardio- and renal protective effects may be demonstrated in this population [20,23,29,31].

Studies with SGLT2i performed in KTRs are much less potent [76] in terms of the number of patients included or the quality of the study design. For this reason, no benefits in terms of patient or graft survival have been demonstrated with its use to date, at least not as strongly as in the general population. However, the benefits published in these studies are also aligned with those demonstrated in the general population [77,78,79,80]. Nevertheless, the most frequently observed side effects are precisely effects that could be potentially harmful to this population profile, such as severe or recurrent urinary tract infections and impaired kidney function [81,82]. Table 3 and Table 4 summarize the effects observed in the studies conducted to date.

Small studies were initially conducted, consistently in case series [83,84,85,86,87,88,89] or a retrospective study with a small sample [91]. Halden et al. [86] performed a single clinical trial with a small population (n = 44), and Schwaiger et al. carried out a small prospective observational study [26]. These initial studies demonstrated a benefit in glycemic control, weight loss, and blood pressure reduction. In more recent studies, such as the multicenter study published by S-Fructuoso et al., that included 339 patients on SGLT2i, in addition to confirming these initial findings, other beneficial effects were observed, notably reduced proteinuria, increased hematocrit, increase in serum magnesium, or decrease in uric acid [93]. Finally, Lim et al. led a retrospective study, with a larger sample (no SGLT2 n = 1857; SGLT2 n = 226), with a control group and a prolonged follow-up time [94]. This publication was the first to demonstrate the benefits of SGLT2 in terms of survival or renal protection in this population. This study showed that the risk of the composite of all-cause mortality, death-censored graft survival, or serum creatinine doubling was significantly lower in KTRs with SGLT2i than in those without SGLT2i (HR: 0.43; 95% CI, 0.24–0.78; *p* = 0.006) [91]. In addition, favorable results were also obtained with the use of SGLT2i in terms of graft survival (HR, 0.34; 95% CI, 0.12–0.95; *p* 0.040) and the progression of creatinine deterioration (HR, 0.41; 95% CI, 0.22–0.77; *p* 0.005). Mortality outcomes were less conclusive (HR 0.24; CI 0.006–0.99; *p* 0.049), as the results were not statistically significant in all the models used [94]. Therefore, more quality studies are required to analyze these benefits in KTRs.

Regarding the adverse effects, urinary tract infections were the most frequent (14–20%) [85,87,88,91,92,93] and those that more frequently forced the withdrawal of the drug (3–5%) [92,93] (Table 4). However, the frequency of these urinary tract infections (UTIs) were also described as similar to that described in other series of transplanted patients with diabetes [93]. Female sex and recent UTIs prior to the onset of SGLT2i could be considered risk factors for these complications [93]; however, hygienic measures and home monitorization with urine dip sticks could be useful in avoiding these in low-risk patients [22,93].

Acute renal failure could be another disadvantage of these drugs, which in some cases forces their withdrawal and in others could delay or discourage their initiation [92]. Some studies have shown unchanging renal function despite the onset of SGLT2i, [81,84,86,87,89,90], while other studies showed an early impairment of renal function (eGFR-dippers) (18–14%), but followed by later stabilization [85,88,93] or an even slower decline in renal function with respect to the control group [94].

In the case of measures aiming to minimize the renal failure associated with the onset of SGLT2i it will probably be interesting to identify those patients at risk of being eGFR- dippers, because this condition probably could be reversible, such as volume depletion, ischemic grafts, high levels of CIN, tendency towards hypotension, septicemia, and other conditions that promote impaired renal function [20,23,76,82,94]. In these cases, measures to alleviate these initial contraindications should be considered (such as the stabilization of renal function, monitoring tacrolimus levels, adjustment of antihypertensive or diuretic, etc.) in order to optimize the conditions of patients so that they could benefit from SGLT2i without risk [22,94].

### 4.2. Potential Benefits of the Use of SGLT2I in General and Renal Transplant Populations

The benefits demonstrated for the use of SGLT2i have to date been related to the control of the metabolic syndrome (reduction in glycemia, weight, blood pressure or albuminuria) [95], but it is difficult to attribute such wide-ranging effects as reduced mortality solely to clinical metabolic syndrome. In fact, it is believed that these drugs may have benefits that ultimately contribute to the reduction in oxidation stress, tissues inflammation, and fibrosis, which would explain these important benefits [68,69]. These protective effects are probably due to several interrelated mechanisms, which turn SGLT2i into “reliever drugs”, because they minimize stressful and toxic mechanisms for cardio and renal parenchyma, thus reducing the progression of chronic heart and kidney disease.

**Figure 4:**
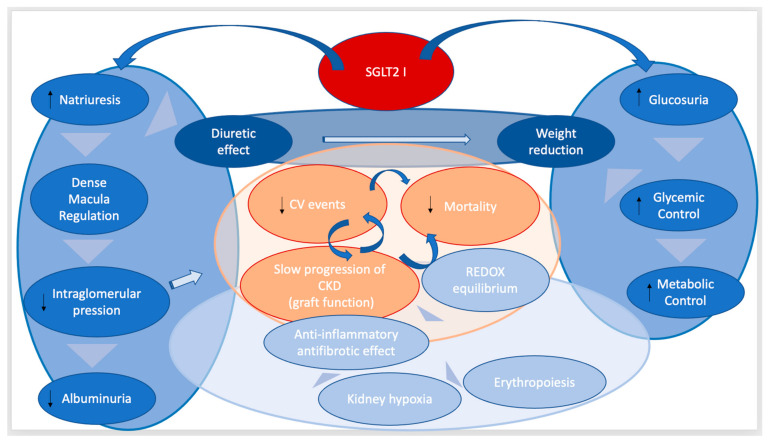
Potential benefits of SGLT2i in CKD and renal transplants. SGLT2i have a direct effect on the tubular co-transcarrier that increases natriuresis and glucosuria. Indirectly, increased natriuresis has been shown to decrease glomerular hyperfiltration by regulating the dense macula. This effect leads to a decrease in renal hyperfiltration and albuminuria, which has been historically associated with a slowing of the progression of CKD. Additionally, this increase in natriuresis results in a diuretic effect. In addition, another direct effect of SGLT2i is that of the reduction in glucosuria reabsorption, which means better glycemic control and a better control of the metabolic profile thanks to the effects also demonstrated by these drugs (weight loss, improved lipid profile) (effects represented in deep blue). The most important demonstrated benefits of these drugs are the reduction in the risk of cardiovascular events, reduced mortality from cardiovascular and renal causes, and the slowing of the progression of CKD (effects are represented in orange). However, these great benefits may in part be ascribed to a beneficial balance between oxidant and antioxidant pathways that are associated with anti-inflammatory or antifibrotic effects or even an erythropoiesis stimulation (effects are represented in light blue).

The main potential mechanisms attributed to SGLT2i to achieve the observed reno-protective effects are summarized in terms of their capacity to reduce glomerular hyperfiltration, renal hypoxia, oxidative stress, and autophagy, as well as stimulate erythropoiesis. The minimization of these processes would lead to the reduction in inflammation and consequently, to the reduction in fibrosis [69] (Figure 4). In fact, it is considered that these mechanisms are not limited to the kidney, but that other organs are also susceptible to these benefits (heart, brain). This could explain the cardiovascular protective effects and those related to the reduction in mortality [68].

One of the most important effects of SGLT2i is the regulation of tubuloglomerular feedback mitigating hyperfiltration through the inhibition of glucose and sodium reabsorption in the proximal tubule via the SGLT2 transporter. This effect decreases glomerular hyperfiltration, thus potentially slowing the progression of glomerulosclerosis [96,97]. Renal hypoxia is a characteristic feature of CKD and has been implicated in the development and progression of kidney disease [95]. Renal status requiring additional ATP (such as hyperfiltration) increases the energy demand and oxygen consumption, predisposing the kidney to hypoxia [69,96]. The inhibition of this ATP-dependent transport by SGLT2i could improve the balance between oxygen supply and its utilization, thus improving tissue oxygenation in the kidney [66].

The REDOX equilibrium system is necessary for normal cell function as they modulate cell-signaling pathways and cellular processes [97]. However, the overproduction of any of oxidative molecules (ROS) is pathological since it can damage macromolecules such as proteins, lipids, or DNA, leading to oxidative stress, energy depletion, impaired energy-dependent repair mechanisms, and cell death [98]. These effects result in mitochondrial dysfunction, endothelial dysfunction, or lipototoxicity, among others [66,99]. Redox system imbalance has been described as an important physiopathological mechanism of kidney diseases [100] that contributes and perpetuates the development of CKD [68]. SGLT2 is involved in numerous REDOX balance pathways, such as the regulation of genes involved in the nitric oxide (NO) pathway [100], fatty acid oxidation, and ketogenesis [68,101], and the reduction in mitochondrial dysfunction [102]. The regulation of these processes could result in renal protection by minimizing inflammation, tubulointerstitial fibrosis, and endothelial injury, as well as reducing lipotoxicity, which could contribute to preventing podocyte effacement, detachment, and podocyte death [103,104,105,106,107].

Another possible mechanism of glomerular protection, in which SGLT2i has been implicated, consists of the activation of cellular autophagy. It would seem that SGLT2i activates autophagy in podocytes, which might prevent mesangial expansion and reduce albuminuria [103]. Although this remains to be demonstrated, it is reasonable to hypothesize that SGLT2i therapy would improve autophagy and restore the balance to the mitochondrial equilibrium [69].

The stimulation of erythropoiesis is also among the described benefits of SGLT2, which occurs through increased levels of bone marrow-derived hematopoietic cells and the suppression of hepcidin levels [28]. These actions might be expected to reduce hypoxic stress, which, in the long term, could prevent or slow the progression of CKD [1].

All of the previously described mechanisms ultimately contribute to inflammation and then to renal fibrosis. Therefore, the use of SGLT2i could contribute to alleviating these mechanisms of lesion contributing to CKD [66,102]. In addition, small experimental studies support a direct anti-inflammatory and antifibrotic effect, since modest reductions in circulating anti-inflammatory markers (IL-6, TNF, and IFNγ) [108] or antifibrotic markers (TGFβ, collagen, or reduced cardiac fibrosis in mouse models) [109] have been observed with the use of these drugs.

Based on findings in the general population and studies in KTRs, the use of SGLT2i can be of great benefit to this population. It seems essential to fully understand these medications to be able to use them in most patients, but their condition must be optimized to minimize risks and avoid their removal. However, it is also essential to identify those patients in whom its use may be harmful and, therefore, discard its initiation. It is also important to note that SGLT2i are associated with a profile of benefits that are especially important in KTRs (and possibly less important among the general population), such as the decrease in uric acid [89,90] or the increase in serum magnesium [85,91,93] and hemoglobin [83,85,93], since these patients frequently present with hypomagnesemia with difficult management, hyperuricemia, and anemia [110].

**Figure 5:**
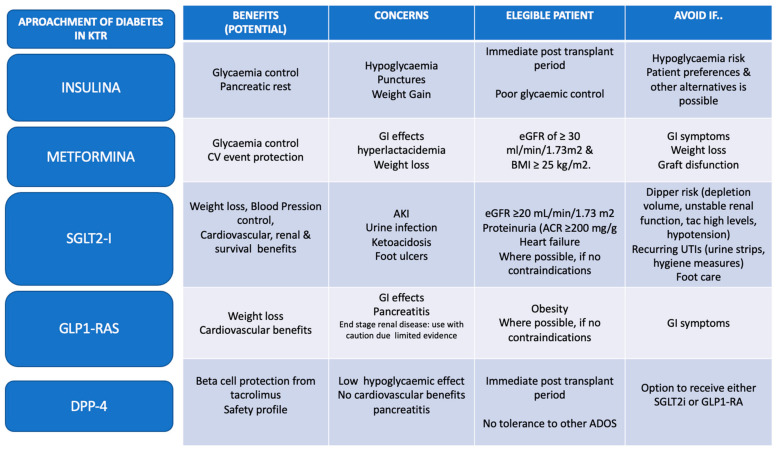
Approaches to diabetes in Kidney Transplant Recipients. Summary of recommendations for the management of diabetes in kidney transplant patients. The main goals of treatment include lifestyle, control of blood glucose, lipids and hypertension, and the use of the currently available therapeutic arsenal. Ultimately, the main goal of diabetes treatment is to minimize cardiovascular events and to improve graft and patient survival. Based on these premises, the use of available drugs will be personalized for each patient in order to achieve the maximum benefit attributed to each drug while minimizing the associated adverse effects. In the case of the use of SGLT2i, it is recommended that concomitant treatment be modified, and that monitoring measures are implemented to optimize patient status and minimize potential adverse effects Adapted of [22,112,113].

Based on the potential effects and mechanisms, it is important to highlight that the combination of SGLT2i and GLP1-RA could be of interest in diabetic renal transplant patients, since this combination would exploit the beneficial effects of both drugs. GLP1-RA increases insulin secretion and glucagon inhibition and thus decreases endogenous glucose secretion, counteracting the increase that SGLT2i could produce. In addition, the hypoglycemic effect of SGLT2i is discrete and the concomitant use of GLP1-RA could enhance the effects of glycemic control and cardiorenal protection, since the mechanisms are different and complementary [111]. Figure 5 summarizes the main advantages and disadvantages associated with the most commonly used antidiabetic drugs today and the possible indications and contraindications to be considered in routine clinical practice.

## 5. Conclusions

In the last decade, the management of diabetes and renal failure has changed thanks to the appearance of new drugs that have benefits in terms of survival and cardiorenal protection. Renal transplant patients are a population that could greatly benefit from the advantages of new antidiabetic oral drugs such as SGLT2i and GLP1-RA. Therefore, it is important to understand the characteristics of these drugs in order to select those appropriate for the patient to benefit from these drugs without being negatively affected by adverse side-effects. In addition, it would be interesting to adapt other concomitant treatments so that patients can improve their condition and safely receive these drugs and thus benefit from their effects in the medium term [19]. The combination of SGLT2i and GLP1-RA could be of interest in renal transplant diabetic patients, since their combined effects on weight and glycemic control as well as cardiorenal protection are produced by different and complementary mechanisms. The close monitoring of patients could allow the use of these drugs. However, better studies are still needed to confirm these benefits in the renal transplant population.

## Figures and Tables

**Table 1:** Diabetic risk factors associated with the kidney transplant population. T2DM, type 2 diabetes mellitus; CIN, anticalcineurin inhibitors; mTOR, mammalian target of rapamycin; HCV, hepatitis C virus; CMV, cytomegalovirus.

Non-Related to Renal Transplant	
Older age	Obesity
African American ethnicity	Hispanic ethnicity
Insulin resistance	Prediabetes
Sedentary lifestyle	Family history of T2DM
Male	
**Related to Renal Transplant**	
CIN	Corticosteroids
mTOR inhibitors	HLA mismatch
HCV	CMV
HCV risk (D+/R−)	CMV risk (D+/R−)
Acute rejection	Deceased-donor Kidney

**Table 2:** More relevant studies of GLP1-RA in SOT recipients. CS, case series; eGFR, estimated glomerular filtration rate; HbA1c, glycated hemoglobin; PS, prospective study; RCT, randomized controlled trial; NA, not available; RS, retrospective study; KTR, kidney transplant recipient; dula, dulaglutide; lira, liraglutide.

Author, Year, Study Type	AIM	n/Follow-up	Glycemic Control ΔHbA1c (g/dL) Mean (±SD)/Median (IQR) ΔGlycemia (mg/dL) Mean (±SD)/Median (IQR)	Body Weight Weight (KG) Mean (±SD)/Median (IQR)	Effect on Renal Function ΔeGFR (mL/min/1.73 m) Mean (±SD)/Median (IQR)	Seric Levels of Tacrolimus	Side Effects
Pinelli et al., 2013 [40] CS	Evaluate short-term effects of liraglutide in KTR with PTDM	5 21 days	ΔHbA1c: NA ΔGlycemia: no changes	ΔKG: −2.1 kg (±1.3)	NA	No changes	Nausea, headache, pain in the puncture site, weakness
Krist et al., 2014 [39] RS	GLP-1 RA in SOT recipients with DM	20 (7KTR) 12 months	ΔHbA1c: −0.02% (±2.0) ΔGlycemia: NA	ΔKG: −7.25kg (±3.4)	No change	No changes	NA
Cariou et al., 2015 [41] RS	Liraglutide, pancreas or pancreas–kidney transplant recipients	6 (0KTR) 6 months	ΔHbA1c: −0.8%/6.9% (6.6–8.4%) ΔGlycemia: NA	ΔKG: −2 kg	No change	No change in doses	Nausea, diarrhea, vomiting
Halden et al., 2016 [42] SCT	Insulinotropic and glucagostatic effects of GLP-1 on KTR with or without DM	24 NA	ΔHbA1c: NA ΔGlycemia: No changes	NA	NA	NA	NA
Liou et al., 2018 [43] RS	Liraglutide in KTR with DM	7 19.4 ± 7.6 months	ΔHbA1c: −1.9%/(10.04 ± 1.61 to 8.14 ± 0.8) *p* < 0.031 ΔGlycemia: −62 mg/dL/(228.6 ± 39.1 to 166 ± 26.6) *p* < 0.103	ΔKG: −2.1 to −3 kg (78.0 ± 7.8 to 75.1 ± 9.1) *p* < 0.032	ΔeGFR: +8.8 mL/min/(67.66 18.69 ± to 76.53 ± 18.6) *p* < 0.024	No changes	Vomiting, nausea, headache, dizziness, pain in the puncture site
Singh et al., 2019 [44] RS	Liraglutide and dulaglutide in SOT recipients with DM	81 (63 dula, 25 lira) 6–24 months	ΔHbA1c: −8.4% dulaglutide (*p* = 0.49) ΔHbA1c: +2% liraglutide (*p* = 0.49) ΔGlycemia: NA	ΔKG: −5.2% dulaglutide (*p* < 0.03) ΔKG: −0.89% liraglutide (*p* < 0.03)	ΔeGFR: +15% dulaglutide, −ΔeGFR:8% liraglutide	No change in doses	Nausea, vomiting diarrhea, abdominal pain, hypoglycemia, cholelithiasis
Thangavelu et al., 2020 [45] RS	GLP-1RA in SOT recipients with DM	19 (7KTR) 12 months	ΔHbA1c: −0.75% (−1.55 to 0.5) ΔGlycemia: NA	ΔKG: −4.86 Kg (−7.79 to −1.93) *p* < 0.05	No changes	No changes	Gastrointestinal effects
Kukla et al., 2020 [46] RS	GLP-1RA in KTR with hyperglycemia	17 (14KTR) 12 months	ΔHbA1c: −0.4%/(−1.05 to 0.55) *p* < 0.07 ΔGlycemia: −8.5 mg/dL/(−33 to 28) *p* < 0.7	ΔKG: −0.95 kg/(−7.2 to 1.0) *p* < 0.2	No changes	No changes	Gastrointestinal, pancreatitis (n = 1)
Yugueros et al., 2021 [47] RS	Effects of new antidiabetic drugs in KT	15 28 months	ΔHbA1c: −0.7%/(5.8–8.2% to 5.3–8.1%) IQR at 12 months *p* < 0.96 ΔGlycemia: NA	ΔKG: −1.7 kg/m^2^/(29.7–35.5 to 27.6–31.6) IQR at 12 months *p* < 0.1	No changes	No changes	Weakness
Kim et al., 2021 [48] RS	Effects of dulaglutide specially in HbA1C	37 6 months	ΔHbA1c: No difference ΔGlycemia: NA	ΔKG: −4.92 kg (*p* < 0.001)	No data	No change in doses	Gastrointestinal, hypoglycemia in three patients (still use insulin)
Vigara et al., 2022 [49] RS	Effectiveness and safety of GLP1-RA in a cohort of KTR	40 6–12 months	ΔHbA1c: −5% (6–7.4%) IQR *p* < 0.18 ΔGlycemia: NA	ΔKG: −3 kg (±15.7) *p* < 0.41	ΔeGFR: +3.5 mL/min (±15.7) *p* < 0.3	No changes	Gastrointestinal effects
Sweiss et al., 2022 [50] RS	Single-center evaluation of safety/efficacy of GLP1-RA in SOT	118 (83 KTR) 3–12 months	ΔHbA1c: −0.8%/(−0.2–−1.7) IQR *p* < 0.0001 ΔGlycemia: NA	ΔKG: −0.2 kg (±16) *p* < 0.0001	ΔeGFR: +5 mL/min (0–13) IQR *p* < 0.0001	No data	Nausea, vomiting, diarrhea, and pancreatitis

**Table 3:** Studies of SGLT2i in KTR. CS, case series; eGFR, estimated glomerular filtration rate; HbA1c, glycated hemoglobin; MC: multicentric study; PS, prospective study; RCT, randomized controlled trial; SBP, systolic blood pressure; SCr, serum creatinine; UTI, urinary tract infection; UPCR, urine protein creatinine ratio; NA: not available; RS: retrospective study; RCT, randomized controlled trial; KT, kidney transplant; PSM: propensity score matched.

Author, Year, Study Type	n/Drug/Follow-up	Glycemic Control ΔHbA1c % (g/dL) Mean (±SD)/Median (IQR)	Body Weight (KG)/(BMI) Mean (±SD)/Median (IQR)	Blood Pressure (SBP or DBP, mmHg) Mean (±SD)/Median (IQR)	Uric Acid (mg/dL) Mean (±SD)/ Median (IQR)	Anemia Hemoglobin (mg/dL), Mean (±SD)/Median (IQR). Hto (%)	Magnesemia (mmol or mEq) Mean (±SD)/Median (IQR)
Rajasekeran et al., 2017 [83] CS	(KT 6) Cana 8 months	ΔHbA1c: –0.84 ± 1.2 *p* = 0.07	ΔKG: –2.14 ± 2.8 *p* = 0.07	ΔSBP: −6.5 ± 10.8 *p* = 0.13) ΔDBP: −4.8 ± 12 *p* = 0.30	NA	ΔHto: +1.6 *p* = 0.08	NA
Shah et al., 2019 [84] PS	25 Cana 8 months	ΔHbA1c: −1.1 (from 8.5 ± 1.5% to 7.6 ± 1%) *p* < 0.05	ΔKG: −2.5 (from 78.6 ± 12.1 to 76.1 ± 11.2)	ΔSBP: −8 (from 142 ± 21 to 134 ± 17)	NA	NA	NA
Schwaiger et al., 2019 [85] P I	14 Empa 10 12 months	ΔHbA1c: +0.4 (from 6.7 ± 0.7 to 7.1 ± 0.8%) *p* = 0.03	ΔKG: −1.6 (from 83.7 ± 7.6 to 78.7 ±7.7) at 4 weeks *p* = 0.06	ΔDBP: −10 (from 86 ± 14 to 76 ± 11) at 12 months *p* = 0.02	ΔUA: −1.8 From 7.7 (6.7–9.4) to 6.9 (5.5–7.3) at 12 m *p* < 0.005	ΔHto: +1.1% (from 38.8 ± 5.6 to 39.9 ± 5.4) at 4 weeks *p* = 0.06	ΔsMg: +0.07 (from 0.70 ± 0.09 to 0.77 ± 0.11) at 12 months *p* = 003
Halden et al., 2019 [86] RCT	22/22 Empa 10 6 months	ΔHbA1c: −0.2 (from −0.6 to −0.1) vs. 0.1 (from −0.1 to 0.4) *p* = 0.025	ΔKG: −2.5 (from −4 to −0.05) vs. +1.0 (from 0.0 to 2.0) at 12 months *p* = 0.014	ΔSBP –5 (from −12 to 1) vs. 2 (from −6 to 8) *p* = 0.06	NA	NA	NA
Mahling et al., 2019 [87] PS	10 Empa 12 months	ΔHbA1c: −0.2 From 7.3 (6.4–7.8) to 7.1 (6.6–7.5) *p* > 0.05	ΔKG: −1 (from −1.9 to −0.2) *p* > 0.05	ΔSBP: −3 (36.3–0.8) *p* > 0.05	NA	ΔHto: +2.5% (0.8–4.1) *p* > 0.05	NA
Attallah and Yassine, 2019 [88] CS	8 Empa 25 12 months	ΔHbA1c: –0.85 At 3 m (then sustained) *p* > 0.05	ΔKG: –2.4 kg (from 76.8 ± 7.4 to 74.94 ± 7.4) at 12 months *p* > 0.05	ΔSBP −4.2. at 3 m *p* > 0.05	NA	NA	NA
Kong et al., 2019 [89] PS	42 Dapa 15 12 months	ΔHbA1c: −0.6 (from 7.5 ± 1.1% to 6.9 ± 0.8%) *p* < 0.01	ΔKG: −1.6 (from 69.6 ± 12.5 to 68.0 ±14.0) *p* < 0.01	No significant ΔHTD: −35.89% (Kong)	NA	NA	NA
AlKindi et al., 2020 [90] CS	8 Empa/Dapa 12 months	ΔHbA1c: −1.93 (from 9.34 ± 1.36 to 7.41 ± 1.44) *p* < 0.05	ΔBMI: −5.3 (from 32.74 ± 7.2 to 27.4 ± 4.2) at 6 months *p* < 0.05	ΔSBP: −11 (from 135 ± 9.59 to 126.43 ± 11.46) *p* < 0.05	NA	NA	NA
Song et al., 2021 [91] RS	50 Empa/Cana/Dapa 6 months	ΔHbA1c −0.53 (±1.79) *p* = 0.118)	ΔKG: −2.95 kg (±3.54) *p* < 0.001	NA	NA	NA	ΔsMg: +0.13 ± 1.73 at 3 months *p* = 0.004
Lemke [92] RS	39 Empa/Dapa/Cana 12 months	ΔHbA1c: −0.6% (−1.2–0) at 3 m *p* 0.013 ΔHbA1c: −0.4% (−1.4–0.1), p *p* = 0.016 at 12 months	ΔKG: −1.6 kg (0–2.7) *p* = 0.11	NA	NA	No differences	NA
Sanchez-Fructuoso et al., 2022 [93] MC	339 Empa/Dapa/Cana 12 months	ΔHbA1c: −0.36. From 7.56 (7.41–7.71) to 7.20 (7.05–7.35) at 6 m. *p* < 0.05	ΔKG: ─2.22. From 81.5 (79.4–83.6) to 79.3 (77.2–81.4) *p* < 0.005 at 6 months	ΔSBP: −4.63. From 137 (135–139) to 132 (130–134) *p* < 0.005 at 6 months	ΔUA: −0.44 From 6.18 (5.98–6.38) to 5.74 (5.55–5.93) *p* < 0.005 at 6 m	Δhemoglobin: +0.15 (0.18–0.11) *p* < 0.001 at 6 m	ΔsMg: +0.15. From 1.61 (1.57–1.66) to 1.76 (1.72–1.80) *p* = 0.001 at 6 m

**Table 4:** More relevant studies of SLGT2i transplant recipients, and the results in terms of renal function and side effects. CS, case series; eGFR, estimated glomerular filtration rate; HbA1c, glycated hemoglobin; mo: months; PS, prospective study; RCT, randomized controlled trial; SBP, systolic blood pressure; SCr, serum creatinine; UTI, urinary tract infection; UPCR, urine protein creatinine ratio; NA, not available; RS, retrospective study; RCT, randomized controlled trial; KT, kidney transplant; PSM, propensity score matched.

Author, Year, Study Type, Follow-up Time	Basal eGFR Median (mL/min)	Effect in Renal Function (eGFR mL/min/1.73 m) Mean (±SD)/Median (IQR)	Proteinuria (uPCR) (g/d)/(uACR) (mg/g) Mean (±SD)/Median (IQR)	Adverse Events
Rajasekeran et al., 2017 [83] CS n 6. 8 mo	78.6 ± 18.2	**No differences** (*p* 0.30) −4.3 ± 12.2	NA	Cellulitis (n = 1) Hypoglycemia (n = 1)
Shah et al., 2019 [84] PS n 25. 8 mo	86 ± 20	**No differences** (*p* > 0.05) ΔeGFR: −3 (from 86 ± 20 to 83 ± 18). At 6 m AKI in 1 patient	NA	None reported
Schwaiger et al., 2019. [85] p I n 14. 12 mo	55.6 ± 20.3	**Decrease and then stabilize** ΔeGF: −8.1 (from 55.6 ± 20.3 to 47.5 ±15.1) at 4 w (*p* 0.008) ΔeGF: −2.1 (from 54.0 ±23.8 to 53.5 ±13.3) at 12 m (*p* 0.093)	ΔuACR: −25 (from 87 (41–552) to 62 (28–348) At 4 w, *p* 0.43 (sch) ΔuACR: −73 (from 289 (190–808) to 216 (137–585). At 2 w (*p* 0.43)	UTI (n = 5) Balanitis (n = 1) pneumonia (n = 1)
Halden et al., 2019 [86] RCT n 44. 6 mo	E: 66 ± 10.5 *p*: 59 ± 9.5	**No differences** ΔeGFR: –3 (from −7 to 0) versus −1.0 (from −2.8 to 0.75) *p* 1.000	NA	UTI (n = 3) genital yeast infection (n = 1) urosepsis (n = 1)
Mahling et al., 2019 [87] PS n 10. 12 mo	57 ± 19.3	**No differences** ΔeGFR: stable. 57 (47–73) mL/min AKI in 1 patient	NA	UTI (n = 2) AKI (n = 1) Diabetic ulcer (n = 1) Tiredness (n = 1) AKI(n = 1)
Attallah and Yassine, 2019 [88] CS n 25. 12 mo	NA	**Decrease and then stabilization** ΔsCr: +11 (from 88.5 mmol/L to 99.5 at 1 m (*p* > 0.05) ΔsCr: +1.5 (from 99.5 to 96.5) at 12 m (*p* > 0.05)	Δ uPCR: −0.6 g/d. At 12 m.	UTI (n = 2) Nausea (n = 2)
Kong et al., 2019 [89] PS n 42. 12 mo	60.36 ± 17.0	**No differences** ΔeGFR: −1 (from 60.3 ± 17.0 to 59.3 ± 14.5). At 12 m	ΔuACR: No significant change was observed at 12 m	Acute cystitis (n = 3) Weight loss (n = 2)
AlKindi et al., 2020 [90] CS n 8. 12 mo	75.8 ± 13.4	**No differences** (*p* < 0.05) ΔeGFR: −6.07 (from 75.75 ± 13.38 to 69.88 ± 14.70)	NA	UTI (n = 1)
Song et al., 2021 [91] RS n 50. 6 mo	66.7	**No differences** −1 mL/min (−7.5–7) at 3 months *p* 0.8831 1 mL/min (−8–16) at 6 months *p* 0.1478	NA	UTI (n = 7) Genital mycosis (n = 1)
Lemke et al., 2021 [92] RS n 39. 12 mo	NA	Dippers: 23% at 12 m ΔeGF: −1.5 (−8.5–5) *p* 0.47 at 3 months. ΔeGF: −2 (−9.5–0.5) *p* 0.11 at 12 months	NA	UTI (n = 6) AKI (n = 1) Diabetic ulcer (n = 2) Ketoacidosis (n = 1) AKI (n = 2) Hypoglycemia (n = 2)
S-Fructuoso et al., 2022 [93] MCO n 339. 12 mo	58.4 (56.2–60.6)	**No differences** Dippers: 1.8% at 12 m ΔeGF: −2.13 (−3.26, −1.0) at 6 months. *p* < 0.005 ΔeGF: −1.87 mL/min (−1.25, +5.02). *p* = 0.122	Δ uPCR: −230 at 6 mo. and −310 at 12 mo. (from 750 (390–1410) to 520 (270–950) at 6 mo and 440 (230–700) at 12 mo. In patients with basal uPRC > 300 g/g. (*p* < 0.001)	UTI (14%) AKI (1.8%) Genital yeast infection (0.9%) Diarrhea (0.6%) Hypoglycemia (1.2%)
Lim et al., 2022 [94] OR, PSM, n 2083 63 mo	S: 66.9 ± 17.7 C: 68.4 ± 20.1	**Decrease, stabilization, and amelioration** Dippers: 15.6%. ΔeGF: −10% at 1 m. ΔeGF:recovered at 5 m (70 mL/min) No dippers: ΔeGF: stable for the first 5 mo and better after 6 m. *p* 0.005 Graft survival HR, 0.34; 95% CI, 0.12–0.95; *p* 0.040 Serum creatinine doubling HR, 0.41; 95% CI, 0.22–0.77; *p* 0.005	Δ uPCR: The urine PCR decreased significantly in the dipper and nondipper groups after SGLT2i usage *p* < 0.005	NA

## Data Availability

Not applicable.
